# Breaking the Mile Barrier: Established Physiological Determinants, Marginal Gains, and Precision in Elite Performance

**DOI:** 10.3390/sports14100443

**Published:** 2026-10-09

**Authors:** Gerasimos V. Grivas

**Affiliations:** Physical Education and Sports, Division of Humanities and Political Sciences, Hellenic Naval Academy, 18539 Piraeus, Greece; grivasger@hotmail.com

**Keywords:** mile running, world record, endurance performance, running economy, marginal gains, performance technology, athlete monitoring, pacing

## Abstract

The men’s mile world record stood for 27 years before Josh Kerr recorded a provisional world-record time of 3:42.66 in London in 2026, improving Hicham El Guerrouj’s 3:43.13 by only 0.47 s (0.21%). This Perspective examines what changed sufficiently across nearly three decades to advance the observed performance frontier. It argues that the record progression is unlikely to reflect a newly identified physiological determinant or revolutionary training method. The principal requirements of elite mile running, including aerobic power, running economy, oxygen uptake kinetics, anaerobic energy provision, speed reserve, and neuromuscular capacity, were already recognized in the era of the previous record. One plausible interpretation is that contemporary performance systems provide greater opportunities for precision, individualization, and integration in the preparation and expression of established performance determinants. Publicly available accounts of Kerr’s Project 222 illustrate this transition: athlete-specific footwear, race-specific pacing, wearable-derived monitoring, controlled hypoxic exposure, structured recovery, individualized nutrition, and multidisciplinary support were reportedly coordinated around one predefined objective. Available public evidence cannot quantify the contribution of any component or identify a single cause. Kerr’s provisional world-record performance may therefore be interpreted not as evidence of a newly identified physiological determinant, but as an example of how established performance determinants and small refinements in performance support, when systematically aligned, may become meaningful at the extreme end of observed elite performance.

## 1. Introduction

For 27 years, Hicham El Guerrouj’s 3:43.13 remained the defining standard of men’s mile running. Set in Rome in 1999, the performance represented one of the most exceptional achievements in middle-distance running and established a benchmark that resisted decades of progression in training science, sports technology, and competition strategies [1,2]. On 18 July 2026, Josh Kerr ran a provisional world record of 3:42.66 in London, becoming the first athlete to break the 3:43 barrier and improving the previous world record by 0.47 s, equivalent to approximately 0.21% [2,3]. The contrast between the longevity of the record and the small magnitude of the subsequent improvement is historically striking, but record longevity should not be interpreted as evidence that the previous mark represented a fixed performance ceiling. A world record is more appropriately understood as the highest observed performance achieved under a specific set of historical, competitive, technological, and environmental conditions. The persistence of a world record also reflects factors such as competitive opportunity and event frequency, race incentives and scheduling, field quality, pacing arrangements, environmental conditions, the relative competitive priority assigned to the 1500 m and mile, the number of record-specific attempts, and normal day-to-day performance variability.

**Figure 1:**
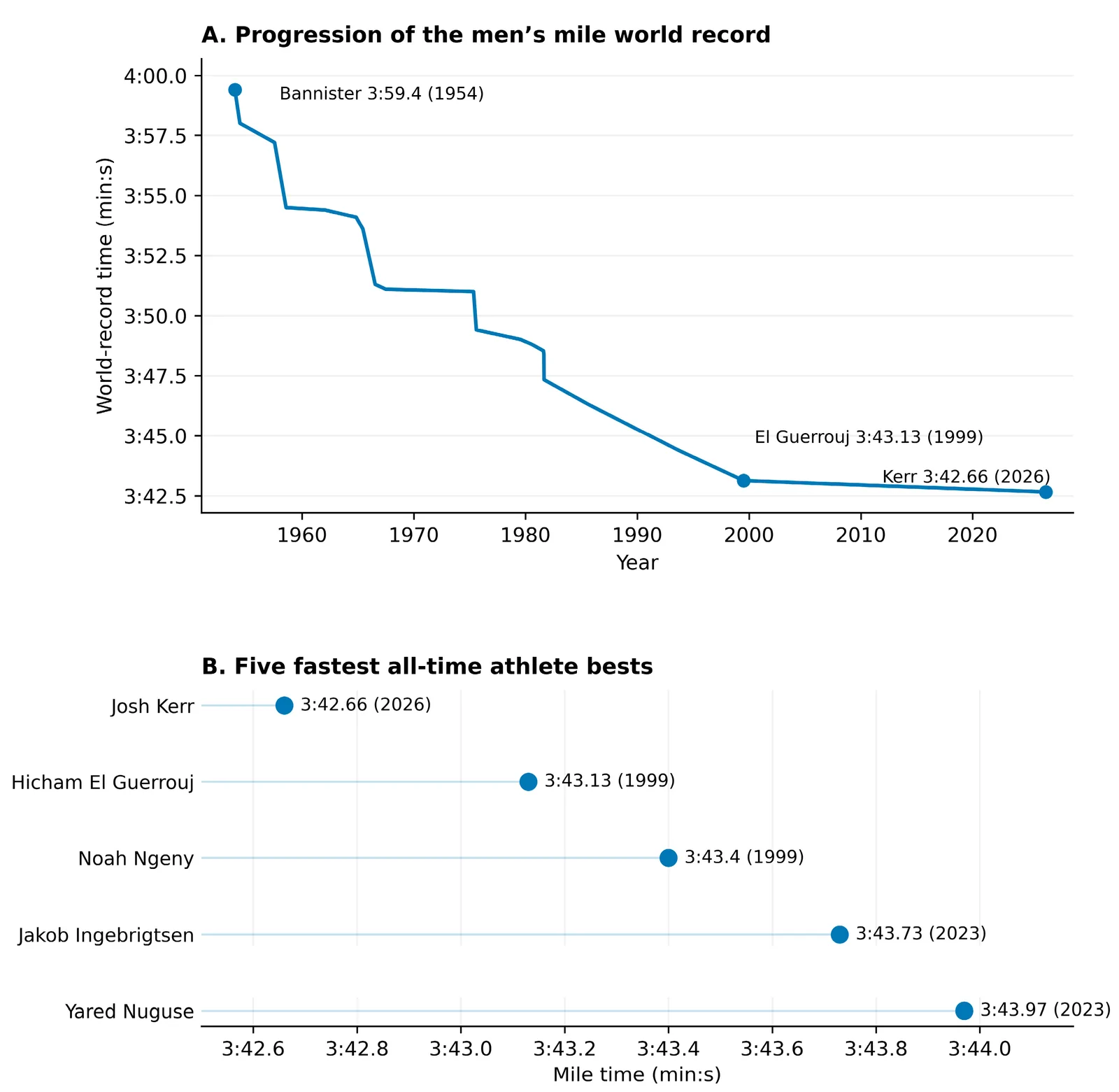
Historical context of elite men’s mile performance. (**A**) Progression of the men’s mile world record from Roger Bannister’s 3:59.4 in 1954 to Josh Kerr’s provisional 3:42.66 in 2026. (**B**) The five fastest all-time athlete bests, illustrating the density of leading performances near the historical world-record level.

The broader performance landscape also provides important context for the record’s longevity. Noah Ngeny ran 3:43.40 in the same 1999 race in which the previous world record was established, while Jakob Ingebrigtsen and Yared Nuguse approached that benchmark in 2023 with 3:43.73 and 3:43.97, respectively. Following Kerr’s 3:42.66 in 2026, the five fastest athletes on the World Athletics all-time list have therefore all recorded performances between 3:42.66 and 3:43.97. Kerr’s own performance trajectory also developed substantially before the record attempt: his previous official track mile best was 3:45.34 in 2024, while his 1500 m championship performance progressed from 3:29.05 at the Tokyo Olympic Games in 2021 to 3:27.79 at the Paris Olympic Games in 2024. These observations place the 2026 performance within a broader evolution of elite middle-distance performance rather than reducing the historical context to the two world-record performances alone (Figure 1).

The mile represents one of the most demanding events in track and field because it requires the integration of aerobic power, running economy, oxygen uptake kinetics, anaerobic energy contribution, maximal speed, and neuromuscular capacity within a single performance lasting approximately four minutes [4,5,6]. Although maximal oxygen uptake (VO_2max_) is an important determinant of endurance performance, elite middle-distance performance cannot be explained by a single physiological variable. Athletes with similar aerobic capacities may achieve different performances because of differences in economy, velocity associated with VO_2max_, oxygen kinetics, anaerobic speed reserve, or the ability to preserve mechanics under fatigue [5,6,7]. Consequently, progression at the highest level is unlikely to result from improvement in one isolated physiological characteristic.

The small numerical margin by which Kerr surpassed the previous world record is historically informative, but it should not be interpreted as the magnitude of Kerr’s physiological or performance development. A world record is a threshold outcome: a performance need only surpass the previous benchmark to establish a new record. However, a small difference between two record performances does not indicate that the physiological, technological, environmental, or training-related differences underlying those performances were themselves small. This concept of marginal gains has become increasingly relevant in high-performance sport, where monitoring, recovery strategies, equipment optimization, and individualized preparation are integrated to improve the probability of achieving peak performance on a specific day [8,9].

Kerr’s performance was also not an unexpected consequence of an uncontrolled or unusually favourable race. The attempt had been announced in advance and specifically prepared as Project 222, referring to a targeted performance of approximately 222 s [2]. The project was publicly described as a record-specific approach in which training, equipment, pacing, environmental preparation, monitoring, and athlete support strategies were coordinated around one competitive objective. Such an approach reflects the increasing ability of modern performance systems to combine information from multiple domains rather than relying on isolated interventions [8,9].

However, the explanation is unlikely to emerge from a direct comparison between Kerr and El Guerrouj. Comparable physiological, biomechanical, nutritional, and training data are unavailable, and the two performances occurred under different competitive, environmental, and technological conditions. Furthermore, El Guerrouj’s 1999 record should not be interpreted as the product of a less advanced era. His performance was achieved with elite pacemaking and highly controlled race execution, while Noah Ngeny finished only 0.27 s behind in 3:43.40, also surpassing the previous world record [1]. Several features associated with contemporary record attempts were therefore already present more than two decades ago.

One hypothesis considered in this Perspective is that contemporary performance systems may provide greater opportunities to measure, individualize, and coordinate the development and expression of established performance determinants. This Perspective therefore evaluates the publicly documented components of Project 222 within that framework, while distinguishing reported practice, general mechanistic evidence, and athlete-specific inference. The principal physiological determinants relevant to elite mile performance were already recognized in the era of the previous world record. Contemporary technologies and performance-support systems provide additional opportunities to measure, individualize, and coordinate preparation across multiple domains. Advances in footwear technology, athlete monitoring, environmental manipulation, recovery management, nutrition, and multidisciplinary support provide new opportunities to reduce uncertainty and optimize the expression of existing physiological capacity [8,9,10,11].

This Perspective proposes that the progression from 3:43.13 to 3:42.66 can be interpreted within an established physiological framework, alongside an evolution in the precision with which performance determinants can be developed, supported, and expressed.

### Evidence Approach

This article was developed as a narrative Perspective rather than a systematic review. Peer-reviewed literature was identified through targeted searches in PubMed, Scopus, Web of Science, and Google Scholar. Initial searches were conducted during manuscript preparation in July and August 2026 and were updated during revision in September 2026. Search concepts included combinations of terms related to mile and middle-distance running, physiological determinants, elite middle-distance training, running economy, anaerobic speed reserve, advanced footwear technology, pacing and visual pacing systems, athlete monitoring, altitude and hypoxic training, recovery and sleep, sports nutrition, and integrated performance support. No formal publication-date restriction was applied. Priority was given to evidence directly relevant to middle-distance performance or to the specific mechanism under discussion, including systematic reviews, meta-analyses, consensus statements, and relevant experimental or observational studies. Foundational studies were retained where they established physiological or training principles relevant to the historical comparison, whereas more recent evidence was prioritized for contemporary technologies and performance-support practices. Peer-reviewed studies were used to evaluate physiological mechanisms and training principles, whereas official event reports, athlete interviews, media reports, and manufacturer or sponsor sources were used only to reconstruct publicly reported components of Project 222. Public-source material was identified through targeted searches using combinations of “Josh Kerr,” “Project 222,” and terms referring to the specific preparation component under investigation. Public-source searches were last updated on 22 September 2026. When multiple accounts were available, official or primary sources were prioritized. Athlete-specific factual claims were cross-checked across sources where possible; when only a single source was available, the claim was explicitly attributed and was not treated as independently verified evidence of effectiveness. Where public accounts conflicted or could not be reconciled, the uncertainty was retained or the claim was omitted. No formal systematic search, risk-of-bias assessment, or meta-analysis was undertaken, and commercial or media sources were not treated as evidence of intervention effectiveness. Accordingly, three levels of evidence are distinguished throughout this Perspective: publicly reported practice, peer-reviewed evidence supporting the general mechanism, and athlete-specific inference. These evidential categories were not treated as equivalent. Statements concerning whether a practice was used were attributed to the relevant source type, including athlete reports, sponsor or manufacturer descriptions, and official race data. Claims concerning measurement validity, physiological mechanisms, adaptation, or performance effects were supported by peer-reviewed evidence where available. Importantly, no causal sequence was inferred in the absence of athlete-specific evidence linking a reported measurement to a decision, that decision to a change in training or recovery, the change to a physiological adaptation, and the adaptation to race performance. Athlete-specific interpretations are treated as hypothesis-generating and are not used to infer effectiveness or causality.

## 2. Established Physiological Determinants and Training Principles

The mile represents a distinctive integration of endurance and speed. Unlike longer endurance events, where maintaining a relatively stable metabolic demand is central, the mile requires the athlete to rapidly attain and sustain a velocity that exceeds the intensity supportable through oxidative metabolism alone. Success therefore depends on the interaction of oxidative and non-oxidative energy provision, speed, and fatigue tolerance throughout the race. Direct experimental estimates of energy-system contribution are not available for the mile itself. Evidence from 1500 m running provides the closest experimental proxy because of the similar event duration and physiological demands, but the two events should not be considered physiologically or tactically identical. The additional distance and duration of the mile may influence pacing distribution, relative energy-system contribution, fatigue development, finishing requirements, and tactical structure. Estimates of approximately 77–84% aerobic contribution have been reported for 1500 m running [4,12,13] and are therefore used here only to contextualize the likely predominance of oxidative metabolism in elite mile performance, rather than as direct estimates for the mile.

Mile performance therefore cannot be reduced to VO_2max_ alone. Although a high VO_2max_ provides the potential for substantial oxidative energy production, elite performance depends on how this capacity interacts with running economy, oxygen uptake kinetics, velocity associated with VO_2max_, anaerobic capacity, maximal sprint speed, anaerobic speed reserve, neuromuscular function, and the ability to preserve mechanical efficiency as fatigue develops [4,5,7,12,13,14,15]. Two athletes with similar VO_2max_ values may achieve different performances because their physiological capacities are expressed differently through economy, speed reserve, fatigue resistance, and race execution. Consequently, elite milers do not conform to a single physiological model. Some athletes may rely relatively more on aerobic power and economy, whereas others may possess greater maximal speed, anaerobic contribution, or neuromuscular qualities [7,15].

This physiological diversity was already evident during the era of El Guerrouj’s record. Research available around the time of the previous world record had already identified neuromuscular characteristics as important contributors to running performance and demonstrated that explosive strength training could improve running economy and performance without increasing VO_2max_ [16,17]. Subsequent systematic-review evidence has further supported the ability of strength training to improve running economy in endurance athletes, including highly trained runners [18]. Later research has refined the measurement of physiological determinants such as aerobic and anaerobic contributions, oxygen uptake kinetics, speed reserve, neuromuscular qualities, and fatigue-related changes; however, these advances have expanded the understanding of elite performance rather than replaced the fundamental physiological requirements of the event.

The same continuity applies to training methodology. Event-specific literature on world-class 800 m and 1500 m runners indicates that preparation combines substantial aerobic running, threshold-oriented training, high-intensity intervals, race-specific repetitions, faster-than-race-pace work, sprint development, and strength or power training, with the relative emphasis varying according to event specialization and athlete profile [7,19,20,21]. Event-specific evidence indicates that 1500 m specialists generally perform greater running volumes and a larger proportion of aerobic training, whereas 800 m athletes place relatively greater emphasis on high-intensity, speed-oriented, strength, power, and plyometric training [21]. Because direct longitudinal evidence concerning world-class mile-specific training is limited, the 1500 m training literature is used here as the closest event-specific proxy. This extrapolation should nevertheless be interpreted cautiously because the mile involves a slightly longer performance duration and may differ in pacing and tactical demands. These training components remain present in contemporary elite programmes, although their distribution, sequencing, and intensity are individualized according to athlete characteristics, training history, and performance goals. Similarly, altitude exposure and tapering were established components of endurance preparation before the contemporary era. Evidence supporting altitude and hypoxic strategies is provided by the previous experimental and review literature [22,23,24], while the scientific basis for precompetition tapering was also well-established [25]. Contemporary approaches may allow for greater individualization of these practices, but they do not represent fundamentally new training stimuli.

The progression of the mile record should therefore not be interpreted as evidence that contemporary athletes possess inherently superior physiological talent compared with previous generations. Athletic performance is the result of complex interactions among biological characteristics, athlete development, coaching, competition opportunities, technology, medical support, and environmental conditions. Taken together, the available evidence indicates that the principal physiological determinants and training principles relevant to elite mile performance were already recognized when the previous world record was set. This conclusion concerns the scientific understanding of the event and should not be interpreted as evidence that the physiological characteristics of elite milers remained unchanged across eras.

## 3. What Changed Around the Athlete? Precision in a Record-Specific Performance System

A prominent feature of the contemporary record attempt was the reported precision with which preparation was organized around a single athlete, a predefined performance target, and a specific competition date [2]. Publicly available accounts indicate that Project 222 incorporated athlete-specific equipment development [26] and monitoring [27], natural and simulated altitude exposure together with structured recovery practices [28,29,30], individualized nutritional support [31,32], and an event-specific assessment of competitive readiness [33]. Race execution was also organized around a predefined target rhythm with designated pacemakers and visual pacing support [2,3]. These sources document practices reportedly implemented during the preparation, but they do not establish the effectiveness of individual components or demonstrate that the integrated preparation system caused Kerr’s provisional world-record performance. Because Project 222 was a deliberately branded and commercially visible undertaking, the prominence of a reported component should not be interpreted as evidence of its importance or effectiveness. Successful outcomes are particularly susceptible to hindsight and survivorship bias, whereby visible elements of the preparation may retrospectively appear more consequential than they were. Public reporting may also underrepresent interventions that were unsuccessful, modified, abandoned, or considered but not implemented. Accordingly, the following sections distinguish between publicly reported practice, peer-reviewed evidence supporting the general mechanism, and athlete-specific inference regarding Kerr’s performance.

For the purposes of this Perspective, precision refers to the degree to which measurement, prescription, timing, individualization, execution, and decision-making are aligned with a predefined performance objective. Greater precision should not be inferred merely from the availability of more data, technologies, or specialist input. It requires that information is sufficiently valid and reliable to reduce relevant uncertainty, refine a decision, improve the match between an intervention and the athlete’s needs, or support more reproducible execution. Increased complexity without evidence of such improvements is therefore not considered greater precision.

### 3.1. Athlete-Specific Equipment and the Evolution of Footwear Technology

Advanced footwear technology represents the most visible technological change surrounding elite running performance. Modern track spikes combine lightweight and resilient materials, altered forefoot geometries, and stiffening elements designed to modify the interaction between the athlete, footwear, and running surface. Experimental studies have reported improvements in running economy and selected performance measures with some advanced spikes compared with traditional designs, although the magnitude of the response varies between models, running velocities, and individual athletes [10,11,34]. However, the external validity of these findings for world-record mile running is limited, because the available experimental studies were conducted in trained runners under controlled conditions and at running velocities below world-record mile pace. These studies therefore provide evidence concerning advanced spikes as a general category, but not the metabolic, biomechanical, or performance effects of the specific spike worn by Kerr, particularly at world-record mile velocity or during curve running. Consequently, the magnitude of any metabolic or performance effect cannot be quantitatively extrapolated to Kerr’s race.

Manufacturer-produced material from Brooks described Kerr’s race-day equipment as including a customized Hyperion 222 spike and an individualized speed suit developed specifically for the record attempt [26]. According to Brooks, the spike was developed through testing related to Kerr’s biomechanics and intended racing rhythm and incorporated a customized carbon-plate system and upper [26]. These descriptions document the manufacturer-reported development and customization process but do not constitute independent validation that the selected configurations were physiologically, biomechanically, or aerodynamically superior to alternative designs. No independent athlete-specific comparative data are available to establish the effects of Kerr’s specific footwear or apparel on running economy, biomechanics, curve running, or performance time at world-record mile velocity.

However, the contribution of footwear cannot be quantified retrospectively. Improvements in running economy do not translate directly into equivalent improvements in mile performance [35,36], particularly during a severe-intensity event where aerobic and anaerobic demands interact and where fatigue influences mechanics throughout the race. The response to advanced footwear also depends on athlete-specific characteristics, including body mass, running mechanics, tendon behaviour, and compatibility between shoe properties and individual movement patterns [10,11,34]. Experimental findings therefore provide a mechanistic rationale for the potential relevance of advanced footwear as a general category, but they do not establish the metabolic, biomechanical, or performance effect of Kerr’s specific spike. Customization should also not be interpreted as optimization in the absence of a predefined outcome, an appropriate comparator, repeatable testing, and evidence that the selected configuration was superior. No athlete-specific metabolic or performance effect can therefore be assigned retrospectively to his competition footwear.

### 3.2. Pacing Support and Race Execution

Race execution has always been central to elite mile performance, and the available race data do not support the assumption that contemporary pacing is inherently more even or more precise than that achieved in earlier world-record performances. In Kerr’s 2026 race, official World Athletics analysis reported quarter-mile splits of 55.55, 56.17, 55.71, and 55.23 s, corresponding to first- and second-half times of 1:51.72 and 1:50.94, respectively [37]. At the standard track checkpoints, Kerr passed 400 m in 55.3 s, 800 m in 1:51.1, 1200 m in 2:46.39, and 1500 m in 3:27.62 [2]. By comparison, El Guerrouj’s 1999 world-record performance was exceptionally even, with reported half-mile splits of 1:51.58 and 1:51.55 [1,37]. These data therefore indicate different successful pacing profiles rather than evidence that one represented intrinsically greater pacing precision. Kerr’s record attempt was nevertheless organized around a predefined target rhythm and incorporated designated pacemakers together with visual pacing support [2,3]. Brannon Kidder reached 400 m in 54.75 s, with Kerr a few strides behind in 55.3 s, and led through 800 m before the pacemakers stepped aside [2]. In this Perspective, pacing support is therefore interpreted as a means of assisting race-plan execution and providing external feedback, rather than as evidence of superior pacing efficiency. Peer-reviewed observational evidence from elite 1500 m competition suggests that Wavelight races may be associated with faster performances and reduced within-race pace variability, but these findings do not establish that Wavelight independently improved Kerr’s performance [38]. Its contribution cannot be separated from athlete capacity, pacemaker assistance, drafting, race intent, field composition, and environmental conditions. World Athletics race analysis further indicated that Kerr covered the measured mile distance without additional distance attributable to race positioning [37]. More detailed information concerning Kerr’s exact position relative to the visual pacing lights, athlete-to-pacemaker separation, and complete environmental measurements was not available from the identified authoritative sources and was therefore not inferred. Accordingly, visual pacing technology is treated here as a reported component of the record attempt rather than as an independently effective intervention.

### 3.3. Monitoring and Individualized Decision-Making

Another major development concerns the quantity and resolution of information available to contemporary athletes and support teams. Publicly available sponsor and manufacturer material reported that Kerr’s preparation incorporated wearable devices to monitor variables including training load, heart rate, sleep, recovery, and heart-rate variability [27]. The collection of these variables does not itself establish their validity, reliability, practical usefulness, or influence on athlete-management decisions. Consumer-device estimates of sleep, heart rate, and heart-rate variability should not be considered interchangeable with validated laboratory or clinical measurements [39]. Such information does not directly determine performance; rather, its potential value lies in identifying longitudinal trends, supporting communication between athlete and coach, and informing decisions regarding training progression, recovery, and readiness. The increasing availability of athlete monitoring systems represents a shift from evaluating performance through isolated measurements toward interpreting multiple indicators over time. Importantly, monitoring does not eliminate uncertainty. Heart-rate variability, sleep metrics, and training-load measures are influenced by multiple physiological and contextual factors and require interpretation within the individual athlete’s history rather than as independent predictors of performance [8,9]. Their practical value also depends on sufficiently valid and reliable measurements, an understanding of normal within-athlete variability, meaningful thresholds, contextual interpretation, and defensible decision rules [8,40]. An increasing volume of monitoring data may also complicate interpretation and decision-making when measurement properties, normal variability, and practically meaningful thresholds are uncertain [8,40]. No athlete-specific evidence is available to determine whether any monitored variable directly altered Kerr’s training, taper, recovery, travel, sleep, or competition decisions or improved his performance. The relevance of monitoring within Project 222 should therefore be interpreted as a reported data-collection process with a potential decision-support role, rather than as evidence that monitoring improved decision-making or independently contributed to Kerr’s performance.

### 3.4. Individualized Environmental, Recovery, Nutritional, and Readiness Support

Publicly available athlete, media, and commercial accounts described Kerr’s preparation as incorporating individualized environmental exposure, structured recovery practices, nutritional support, and a race-specific assessment of competitive readiness. Kerr lived and trained at moderate natural altitude in Albuquerque and reportedly used an altitude-controlled bedroom for additional hypoxic exposure for approximately 12 h per day [28,29,30]. However, the available sources do not provide the simulated altitude or inspired oxygen concentration, cumulative hypoxic dose, adherence, timing relative to training, iron status, haemoglobin mass or other haematological outcomes, illness or sleep responses, timing of descent, or evidence that testing resulted in changes to the hypoxic protocol. Accordingly, the altitude-bedroom exposure is presented here only as a reported component of Kerr’s preparation. No athlete-specific change in haemoglobin mass, maximal aerobic power (VO_2max_), running economy, or performance can be inferred, and the available evidence does not permit its physiological contribution to be classified as beneficial, neutral, or potentially disruptive.

Recovery practices were also publicly reported during the preparation period, including planned sleep routines, post-training naps, regular physiotherapy, and designated recovery periods [29,30]. Sleep and recovery are foundational components of elite preparation because they may support training availability, adaptation, health, and readiness across repeated training sessions [41,42]. However, the presence of structured recovery resources does not demonstrate that they produced a measurable physiological or performance benefit in Kerr. No athlete-specific data are available to determine whether these practices altered recovery status, training tolerance, injury risk, or subsequent performance. They are therefore interpreted as supportive practices intended to facilitate consistent training and recovery rather than as independently measurable contributors to Kerr’s provisional world-record performance.

Nutritional support was also publicly reported, including work with a nutritionist and full-time chef and the use of sweat testing to estimate sweat sodium losses and inform electrolyte provision [31,32]. Contemporary recommendations for athletics support individualized and periodized approaches to energy intake, macronutrient availability, hydration, and electrolyte replacement according to training and environmental demands [43,44]. In this context, these practices are best interpreted as supporting training availability, recovery, health, and consistency across the preparation period. In particular, sweat sodium assessment and electrolyte provision have an indirect relationship to a four-minute mile performance and should not be interpreted as an acute ergogenic explanation for the record. No athlete-specific evidence is available to quantify the contribution of these nutritional practices to Kerr’s race performance.

A publicly reported late-stage 1200 m time trial was described as an event-specific readiness check during Kerr’s preparation. Conducted on 3 July 2026 in Albuquerque, New Mexico, at approximately 1626 m of altitude, the session reportedly involved Brannon Kidder pacing Kerr through the first 800 m at approximately 55 s per lap, followed by a 51.88 s final 400 m and a reported total time of 2:42.45 [33,45]. Contemporary reporting described the effort as involving a rolling start and hand timing rather than an official timing system [45]. The performance was therefore not independently verified as an official competitive result. Publicly available sources do not provide sufficient information regarding the preceding training and recovery, complete environmental conditions, footwear, or a formal rationale for selecting this trial over other readiness indicators. Accordingly, the session is interpreted here as a reported event-specific readiness check rather than as a validated predictor of subsequent mile performance.

These practices should not be interpreted as independent explanations for Kerr’s provisional world-record performance. Their relevance within this subsection is more specific: they document how environmental exposure, recovery, nutrition, and event-specific readiness were reportedly managed during the preparation. The broader significance of combining these elements within a single performance system is considered in the following section.

### 3.5. From Multiple Support Practices to an Integrated Performance Framework

The presence of multiple support practices around the same athlete does not, by itself, demonstrate an integrated performance system. For the purposes of this Perspective, integration is defined more narrowly as the coordinated use of information, decisions, and interventions across performance domains in pursuit of a shared performance objective. This interpretation is consistent with broader models of athlete monitoring and multifactorial performance preparation, in which information from different domains is considered in relation to training status, recovery, health, and competition demands [8,9].

Evidence of genuine integration would therefore require more than the coexistence of multiple technologies or support services. It could include documentation that information obtained in one domain altered a decision in another, that support staff worked from shared performance targets, that predefined decision criteria were used to modify preparation, or that trade-offs among training, recovery, health, environmental exposure, equipment, nutrition, and race execution were explicitly managed.

This Perspective therefore proposes a record-specific preparation framework linking measurement, decision, implementation, athlete response, and performance evaluation. Within this framework, the value of a measurement depends on whether it informs a defensible decision, while the relevance of an intervention depends on how it is implemented and how the athlete responds [8,9]. A physiological or performance effect should not be inferred unless the relevant links between measurement, decision, intervention, adaptation, and performance are supported by evidence. Integration, in this sense, refers to coordination across these pathways rather than to the simple accumulation of measurements, technologies, or specialist input.

Publicly available accounts of Project 222 indicate that multiple aspects of Kerr’s preparation were organized around a common record attempt, predefined target pace, and competition date [2]. These accounts document athlete monitoring, equipment development, environmental exposure, recovery support, nutritional support, and race-specific preparation [26,27,28,29,30,31,32,33]. However, the available public evidence does not establish whether information obtained in one domain systematically altered decisions in another, whether formal multidisciplinary decision rules were used, or whether specific trade-offs were actively coordinated across the support team. Project 222 should therefore not be interpreted as proof that a fully integrated performance system, as operationally defined above, was implemented.

The conceptual contribution of the case is consequently narrower. Project 222 provides a publicly documented example through which record-specific preparation can be examined using an explicit integration framework. Established physiological and training determinants form the foundation, monitoring and specialist support may provide information or enabling resources, decisions translate that information into athlete-specific actions, and race execution represents the final performance context in which these processes converge. The value of this framework lies in distinguishing what is documented, what is mechanistically plausible, and what remains unverified (Table 1).

Project 222 is therefore treated here as an illustrative case rather than as evidence that integration itself caused Kerr’s provisional world-record performance. Future investigations of elite record attempts could test the proposed framework prospectively by documenting measurement-to-decision pathways, cross-domain modifications, predefined decision criteria, athlete responses, and subsequent performance outcomes.

## 4. Interpreting a Small Record Margin: Marginal Gains as a Plausible Framework

For the purposes of this Perspective, a marginal gain is defined as a small improvement in a performance-relevant process or outcome, or a small reduction in an avoidable source of performance loss, that is mechanistically plausible and relevant to the demands of the event. A mechanistically plausible effect should not, however, be described as an observed performance gain unless the change is directly measured and can be distinguished from measurement error and normal within-athlete variability. Where these conditions are not met, the term refers only to a potential contribution rather than a demonstrated gain. Reducing a performance loss and producing a positive adaptation are therefore treated as related but conceptually distinct pathways, because both may improve the final performance outcome without necessarily operating through the same physiological, mechanical, or behavioural mechanism.

Accordingly, three evidential levels are distinguished in the present analysis. A demonstrated athlete-specific gain would require direct measurement in Kerr together with sufficient information to interpret the magnitude of change relative to measurement uncertainty and normal within-athlete variability. General mechanistic evidence indicates that an intervention could plausibly influence a performance-relevant determinant but does not establish that such an effect occurred in Kerr. Athlete-specific interpretations based on publicly reported practices are therefore treated as hypothesis-generating unless the relevant measurement, decision, adaptation, and performance links are directly documented.

The historical significance of Kerr’s performance is disproportionate to the numerical magnitude of the record margin. His time of 3:42.66 surpassed the previous record by only 0.47 s, equivalent to approximately 0.21% [1,2]. Importantly, this 0.21% value represents the margin by which Kerr surpassed the previous world record, not his longitudinal improvement as an athlete. Kerr’s previous official track mile best was 3:45.34, recorded in 2024, such that his progression to 3:42.66 represented an improvement of 2.68 s, or approximately 1.19% [48]. A world record is therefore a threshold outcome: it does not require a large transformation in performance, but only a performance that marginally surpasses the previous benchmark. The small record margin therefore describes the threshold by which the previous benchmark was surpassed, but it does not reveal the magnitude, direction, or origin of the underlying differences between the two performances.

Experimental evidence indicates that some advanced track spikes can improve running economy or selected performance measures, although the magnitude varies between models, athletes, velocities, and testing conditions [10,11,34]. Changes in running economy can influence distance-running performance [35]. However, the available footwear evidence was derived from controlled experimental conditions in trained runners and generally at velocities below world-record mile pace. Laboratory-derived metabolic savings therefore cannot be quantitatively translated into a specific improvement in mile time or assigned to Kerr’s performance [36]. The footwear evidence should consequently be interpreted as providing mechanistic plausibility for a potential performance-support role rather than as evidence of a measurable contribution to the 0.47 s record improvement.

The same reasoning applies to race execution. Small deviations from the intended pace may influence race execution, but their contribution cannot be inferred from the 0.47 s record margin itself. The combination of designated pacemakers and continuous visual pacing information may have helped Kerr remain closer to the required rhythm [3,38]. Drafting may also have reduced the energetic cost of overcoming air resistance, particularly at the high velocities required during a record attempt, although the magnitude of this effect depends on the positioning, proximity, and number of pacemakers, as well as the environmental conditions [46,47]. No athlete-specific aerodynamic data are available, and no part of the 0.47 s improvement can be assigned to drafting. Its relevance is simply that even a small mechanically plausible saving could matter at such a narrow performance margin.

The practical relevance of marginal gains lies in whether small changes in performance-relevant processes, including both positive adaptations and reductions in avoidable performance losses, can influence the final expression of performance. At the level of a world-record mile, residual fatigue, pacing variation, equipment mismatch, suboptimal recovery, or small aerodynamic costs may each be insufficient to explain performance independently. Reducing one or more of these limitations could, in principle, influence the final outcome, but the available evidence does not establish that this occurred in Kerr. The marginal-gains framework therefore remains one plausible way of considering how multiple aspects of preparation and race execution might influence elite performance, but the 0.21% record margin cannot be used to infer the magnitude of those effects or to establish that an accumulation of small gains produced Kerr’s performance. A small net difference between the two record performances may coexist with substantially larger positive and negative differences across physiology, training history, technology, race execution, environmental conditions, and other determinants.

## 5. Practical Implications for Coaches and Performance Support Teams

The practical lesson from this Perspective is not that coaches should reproduce a fixed package of technologies or support services. Performance support should begin with an event-specific needs analysis and an assessment of the individual athlete’s current profile. For an elite miler, this requires consideration of the physiological, mechanical, tactical, and training demands of the event, together with the athlete’s training history, health status, recovery capacity, strengths, and identifiable limiting factors. Established training principles, sufficient recovery, athlete health, and consistent training availability should remain the foundation. Additional support should be introduced only when it addresses a clearly defined performance problem or decision rather than because a technology or service is available.

Monitoring should be evaluated according to the quality and interpretability of the information it provides. Before a measure is used to guide training or recovery, coaches and support staff should consider its validity, reliability, typical measurement error, normal within-athlete variability, and, where appropriate, the smallest worthwhile change [8,49]. Single observations should rarely be interpreted in isolation. Longitudinal within-athlete trends, collected under sufficiently standardized conditions and interpreted alongside training context, athlete feedback, and performance data, are generally more informative than isolated measurements or decontextualized thresholds [8,9,50]. Where measurement uncertainty is similar to or greater than the change in practical interest, the data should not be used to justify precise changes in preparation [49,51].

Each additional intervention should also be linked to an explicit decision pathway. Before implementation, the intended purpose, expected response, measurement strategy, time frame for evaluation, and criteria for continuation, modification, or discontinuation should be specified where feasible. This reduces the risk of retrospectively interpreting every observed change as beneficial. If an intervention produces no interpretable response, creates excessive burden, interferes with key training sessions, or introduces adverse effects that outweigh its plausible benefit, its continuation should be reconsidered. Multiple strategies should also not be assumed to have additive effects, because interventions may interact, overlap, or compete for the athlete’s time and adaptive capacity.

The practical value of a support strategy also depends on feasibility. Cost, staff expertise, logistical complexity, athlete burden, travel requirements, data-collection demands, and the opportunity cost of additional testing should be weighed against the expected value of the information or intervention. A technically sophisticated strategy may have little practical utility if it disrupts training, sleep, recovery, or daily routines, or if the resulting information does not alter a meaningful decision. Athlete preference, understanding, and consent should also remain central, particularly when monitoring extends into sleep, recovery, nutrition, or other aspects of daily life.

Finally, multidisciplinary support should complement rather than replace coaching judgement. Effective communication is required so that physiologists, physicians, nutrition practitioners, biomechanists, equipment specialists, and coaches interpret information in relation to a common performance objective rather than generating separate recommendations. The athlete should remain an active participant in this process. Support teams should also evaluate unintended consequences, including data overload, conflicting recommendations, unnecessary changes to successful routines, excessive dependence on technology, or reduced athlete confidence when monitoring signals are ambiguous. The applied priority is therefore not to assemble more technologies or specialists, but to use additional support selectively when it addresses an identified need, produces interpretable information, improves a defensible decision, and does so without disrupting the training process that develops the athlete’s underlying performance capacity.

## 6. Limitations of the Available Evidence

A principal limitation of this Perspective is the absence of published athlete-specific data from Project 222. The available information derives primarily from interviews, media reports, sponsor material, manufacturer descriptions, and wearable-derived observations. These sources are useful for identifying the practices and technologies reportedly included in Kerr’s preparation, but they do not provide independently verified physiological, haematological, biomechanical, nutritional, or training data. Public and commercial accounts may also selectively emphasize novel or marketable aspects of the preparation, while providing limited information about routine training practices, unsuccessful interventions, or decisions that were modified or abandoned. A further limitation is the absence of an appropriate counterfactual. The available evidence does not permit comparison with unsuccessful record attempts, athletes using similar technologies who did not achieve record performances, Kerr’s previous races, periods in which individual components of the reported preparation were absent, or alternative preparation systems. Consequently, the successful outcome cannot be used retrospectively to infer that all visible components were beneficial, that any one component was necessary, or that Project 222 as a whole was superior to other approaches. The possibility of hindsight and survivorship bias is therefore substantial, particularly because unsuccessful, abandoned, or less marketable elements of preparation are less likely to be represented in public accounts. It is therefore not possible to determine whether the reported interventions altered haemoglobin mass, running economy, oxygen uptake kinetics, recovery status, biomechanical efficiency, or any other performance-related variable. Nor can the independent or combined contribution of footwear, pacing support, hypoxic exposure, monitoring, recovery, nutrition, or multidisciplinary support be quantified retrospectively. Interactions among these components further prevent their effects from being separated or added arithmetically. At the time of manuscript preparation, Kerr’s mark also remained subject to formal World Athletics ratification [2]. A direct comparison with El Guerrouj’s 1999 preparation is also not possible. Equivalent information concerning his physiological characteristics, training programme, equipment development, recovery practices, and support environment is unavailable. Differences in the extent of public and commercial documentation between the two eras should therefore not be interpreted as evidence of differences in the quality, sophistication, individualization, or integration of preparation. The two performances therefore cannot be used to establish that Kerr possessed superior physiology or that the contemporary preparation system was definitively more effective.

## 7. Conclusions

Kerr’s provisional world-record performance can be interpreted within the established physiological framework of elite mile running rather than as evidence of a newly identified performance determinant or revolutionary training method. Project 222 provides a publicly documented example of record-specific preparation involving multiple performance-support practices, but the available evidence does not establish that this organization, or any individual component, caused the record. The 0.47 s difference, equivalent to approximately 0.21%, represents the margin by which Kerr surpassed the previous world record rather than the magnitude of any physiological adaptation or performance-support effect. One plausible interpretation is that coordinated preparation may have supported the expression of Kerr’s performance capacity and reduced some sources of performance loss, but the contribution of individual components cannot be established or quantified from the available evidence.

## Figures and Tables

**Table 1:** Evidence framework for publicly reported components of Project 222 and their potential relevance to elite mile performance.

Reported Component	Source Type/Evidence Status	General Mechanistic Rationale	Athlete-Specific Evidence in Kerr	Main Limitation/Uncertainty
Training and record-specific preparation	Official/public reporting documents a predefined record target and competition date [2]. Peer-reviewed evidence on middle-distance training and precompetition tapering [21,25]	World-class middle-distance preparation incorporates aerobic, high-intensity, race-specific, speed, strength/power, and tapering strategies [21,25]	Preparation was organized around a predefined record target; a late-stage 1200 m readiness trial was also reported [2,33,45]	Complete training programme, load distribution, decision rules, and physiological responses are unavailable; the 1200 m trial was not a validated predictor of mile performance
Footwear and apparel	Manufacturer description of Kerr-specific equipment [26]; peer-reviewed experimental evidence [10,11,34]	Advanced spikes may influence running economy, biomechanics, and selected performance measures, with athlete- and model-specific responses [10,11,34]	Brooks reported customized footwear and apparel for the record attempt [26]	No independent metabolic, biomechanical, aerodynamic, or performance effect of Kerr’s specific equipment can be quantified
Pacing support and race execution	Official race data [2,37]; peer-reviewed observational evidence on visual pacing [38]	Pacemaking, visual pacing, and drafting may influence race execution and energetic cost [38,46,47]	Kerr’s splits, pacemakers, and visual pacing support are documented [2,37]	The independent contribution of pacing technology cannot be separated from athlete capacity, pacemaking, drafting, race context, and environmental conditions
Athlete monitoring	Sponsor/manufacturer reporting for Kerr [27]; peer-reviewed monitoring and validation literature [8,39,40]	Monitoring may support interpretation of training response, fatigue, recovery, and readiness when measurements are valid, reliable, and contextually interpreted [8,39,40]	Training load, heart rate, sleep, recovery, and HRV were reportedly monitored [27]	No public evidence shows that a monitored variable altered a specific training, taper, recovery, travel, or competition decision or improved performance
Environmental preparation	Athlete/media reporting [28,29,30]; peer-reviewed altitude/hypoxia literature [22,23,24]	Altitude and hypoxic exposure may influence physiological adaptation, with substantial individual variability [22,23,24]	Kerr reportedly combined natural altitude exposure with additional simulated hypoxia [28,29,30]	Hypoxic dose, adherence, biomarkers, physiological responses, and performance effect are not available
Recovery and nutrition	Media/public reporting [29,30,31,32]; consensus and peer-reviewed evidence [41,42,43,44]	Recovery, sleep, adequate energy availability, hydration, and individualized nutrition support training availability, health, and adaptation [41,42,43,44]	Structured recovery, sleep, nutritional support, and sweat testing were publicly reported [29,30,31,32]	No athlete-specific evidence demonstrates an acute ergogenic effect or quantifiable contribution to the mile performance
Integrated performance framework	Mixed public sources describing multiple domains [2,26,27,28,29,30,31,32,33,37]; peer-reviewed multifactorial framework [9]	Multifactorial preparation may facilitate coordination across training, recovery, health, and competition preparation [9]	Multiple practices were publicly described around a common objective	Coexistence does not establish genuine integration; formal cross-domain decision pathways, trade-offs, and a causal performance effect are not documented

Note. Reported Project 222 components describe practices identified in athlete, sponsor, manufacturer, media, or official sources and should not be interpreted as evidence of effectiveness. General mechanistic evidence refers to peer-reviewed literature. Absence of athlete-specific evidence does not demonstrate absence of an effect; it indicates that the effect cannot be established from the available public record.

## Data Availability

No new data were created or analyzed in this study. Data sharing is not applicable to this article.
